# Identification of a Novel Small RNA Modulating *Francisella tularensis* Pathogenicity

**DOI:** 10.1371/journal.pone.0041999

**Published:** 2012-07-25

**Authors:** Guillaume Postic, Iharilalao Dubail, Eric Frapy, Marion Dupuis, Jennifer Dieppedale, Alain Charbit, Karin L. Meibom

**Affiliations:** INSERM U1002, Université Paris Descartes, Sorbonne Paris Cité, Faculté de Médecine, Paris, France; The Methodist Hospital Research Institute, United States of America

## Abstract

*Francisella tularensis* is a highly virulent bacterium responsible for the zoonotic disease tularemia. It is a facultative intracellular pathogen that replicates in the cytoplasm of host cells, particularly in macrophages. Here we show that *F. tularensis* live vaccine strain (LVS) expresses a novel small RNA (sRNA), which modulates the virulence capacities of the bacterium. When this sRNA, designated FtrC (for *Francisella tularensis* RNA C), is expressed at high levels, *F. tularensis* replicates in macrophages less efficiently than the wild-type parent strain. Similarly, high expression of FtrC reduces the number of viable bacteria recovered from the spleen and liver of infected mice. Our data demonstrate that expression of gene *FTL_1293* is regulated by FtrC. Furthermore, we show by *in vitro* gel shift assays that FtrC interacts specifically with *FTL_1293* mRNA and that this happens independently of the RNA chaperone Hfq. Remarkably, FtrC interacts only with full-length *FTL_1293* mRNA. These results, combined with a bioinformatic analysis, indicate that FtrC interacts with the central region of the mRNA and hence does not act by sterically hindering access of the ribosome to the mRNA. We further show that gene *FTL_1293* is not required for *F. tularensis* virulence *in vitro* or *in vivo*, which indicates that another unidentified FtrC target modulates the virulence capacity of the bacterium.

## Introduction

*Francisella tularensis* is the causative agent of tularemia, a vector- and water-borne disease that affects many mammals, including humans. It is a small, Gram-negative intracellular bacterium that can provoke a potentially lethal disease with an infectious dose as low as 10 bacteria [1]. Central to the pathogenesis of *Francisella* is its ability to invade and multiply in immune cells, such as macrophages, but the bacterium is able to enter a broad range of cells. After uptake, the bacterium resides transiently in a phagosome, but escapes to the cytoplasm where it replicates. The molecular mechanisms of bacterial uptake, phagosomal escape, cytoplasmic replication and how the bacterium evades host immune defenses are not fully understood.

A large number of genes required for virulence have been identified in various genome-wide screens in different strains and subspecies of *F. tularensis* (for reviews see, [2], [3]), but the way by which most of these genes contribute to virulence is not known. The determinant that seems to play the largest role in intracellular multiplication and virulence is the *Francisella* pathogenicity island (FPI), which possibly encodes a type VI secretion system [4], [5]. Generally, strains with mutations in any of the genes encoded in the FPI are attenuated for virulence and some genes have been shown to be essential for phagosomal escape [6]. Other virulence determinants with well-documented roles are the O-antigen and lipopolysaccharide (LPS), siderophores, and the chaperone ClpB (for reviews see [2], [3]).

Several regulatory proteins that are important for virulence have been identified. The three proteins MglA, SspA, and FevR (also called PigR) regulate the same set of approximately 100 genes, including the FPI [7], [8], [9], [10]. These proteins seemingly interact with each other and with the RNA polymerase leading to binding to the promoter region and transcriptional activation of target genes, an event that is dependent on the signal molecule ppGpp [7], [9], [10]. The orphan response regulator PmrA, which is phosphorylated by KdpD, also regulates a large number of genes, including the FPI, but the remaining genes are different from the set regulated by MglA, SspA, and FevR [11], [12]. The MigR protein affects transcription of FPI genes, probably indirectly by controlling transcription of FevR [13]. Finally, inhibition of the sensor histidine kinase QseC leads to decreased expression of several FPI genes [14], strongly indicating that this regulator controls expression of the FPI, directly or indirectly. However, the majority of genes identified in virulence screens are found outside the FPI. At present, very little is known about what controls expression of these, although a number seems to be induced after temperature up-shift and during the intracellular phase [15], [16], [17]. *F. tularensis* expresses few traditional transcriptional regulators. No complete two-component systems exist and only a single alternative σ-factor, a heat shock σ-factor, is encoded in the genome [12], [16]. It therefore seems likely that *F. tularensis* uses other regulatory mechanisms to adapt to the environmental changes it experiences during its lifecycle, in the environment and in the mammalian host.

It is firmly established that RNA transcripts act as regulators of gene expression in all kingdoms of life, including microRNA (miRNA) and small interfering RNA (siRNA) in eukaryotes. In bacteria, some RNA regulators are part of the transcript they regulate, whereas others are small RNAs (sRNAs) that act on independently expressed targets, either a protein or a mRNA [18], [19], [20]. The largest and most extensively studied group is the *trans*-encoded sRNAs that regulate mRNAs by a base-pairing mechanism, often forming short imperfect interactions. In Gram-negative bacteria, many of these *trans*-encoded sRNAs require the RNA chaperone Hfq for stability and/or function. The variety of physiological functions that are regulated by sRNA/Hfq, including virulence in several pathogenic species, is well illustrated by the pleiotropic phenotypes of *hfq* mutants in different bacteria (for reviews, see [21], [22]). Generally, *hfq* mutants are more sensitive to cellular stresses and often exhibit growth defects. We and others have shown that, in *F. tularensis*, deletion of *hfq* affects expression of numerous genes and impacts both virulence and stress resistance, suggesting that sRNAs regulate these functions [23], [24].

Recently we reported the finding of the first two sRNA molecules in the *F. tularensis* live vaccine strain (LVS) [25]. These two RNAs were expressed at relatively high levels, but deleting either gene was not deleterious for the bacterium, *in vitro* or *in vivo*. Here, we present the identification and characterization of a novel sRNA specific to *Francisella* that we refer to as FtrC (for *Francisella tularensis* RNA C). High expression of FtrC reduces intracellular multiplication of *F. tularensis* in macrophages and in organs of infected mice. We furthermore identified a target gene for FtrC regulation, although this target is not involved in bacterial intracellular multiplication. FtrC is the first sRNA shown to modulate the virulence capacity of *F. tularensis*.

## Results

### Identification of a novel *Francisella tularensis* sRNA

**Figure 1:**
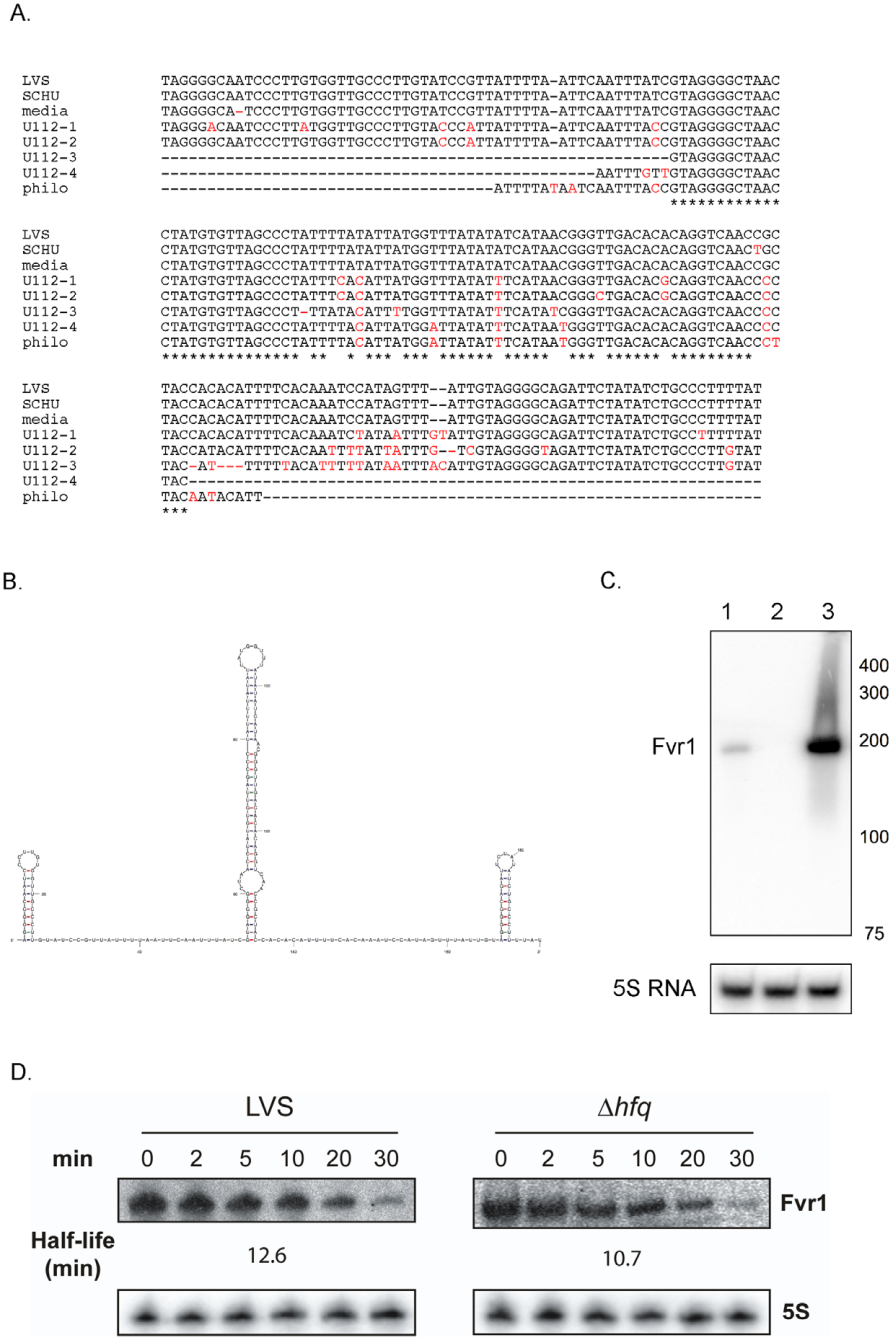
Sequence, structure and expression of *ftrC*. (A) Sequence of *ftrC* in different *F. tulrensis* subspecies. LVS: *F. tularensis* subsp. *holarctica* strain LVS; SCHU: *F. tularensis* subsp. *tularensis* strain SCHU S4; media: *F. tularensis* subsp. *mediasiatica* strain FSC147; U112: *F. tularensis* subsp. *novicida* strain U112; philo: *F. philomiragia* strain ATCC 25071. The numbers after U112 indicate the four different copies of *ftrC*. Nucleotides that are conserved in all strains are marked with an * and those that differ from the LVS sequence are shown in red. (B) Predicted secondary structure of FtrC (using mFold). (C) Northern blot showing FtrC expression in wild-type LVS bacteria (lane 1) and in a strain overexpressing FtrC, LVS/p*ftrC*+ (lane 3). Lack of signal in lane 2 (LVSΔ*ftrC*) confirms absence of FtrC in the deletion strain. Size markers are indicated in nt at right. (D) Northern blot showing the stability of FtrC in LVS and LVSΔ*hfq* strains. Total RNA was isolated at different times (0–30 min, indicated at top) after addition of rifampicin and same amount of each sample was loaded onto gel. The estimated half-lifes (in min) are indicated. 5S RNA served as loading control.

Using a direct cloning and sequencing strategy [25], we have discovered a new sRNA, FtrC encoded on the minus strand between genes *FTL_0777* and *FTL_0778*. The gene flanking *ftrC* on one side encodes a hypothetical protein (FTL_0777) and the other is a pseudogene (*FTL_0778*). To determine the transcription start and termination site of the RNA encoded by *ftrC*, we performed 5′ and 3′ rapid amplification of cDNA ends (RACE). This assay demonstrated that FtrC is 196 nt in length (Fig. 1B) and that the gene encompasses coordinates 765475–765280 in the *F. tularensis* LVS genome. Using BLAST searches, we identified *ftrC* homologues in other *F. tularensis* strains and in other subspecies, each exhibiting 90–100% identity with the LVS gene (Fig. 1A). A *ftrC* homolog was also found in *Francisella philomiragia*, but this homolog only contained the central part of *ftrC*. We did not identify homologues in any other bacterial species by BLAST search and did not find any match to the RNA families in the Rfam database (http://rfam.sanger.ac.uk). This indicates that FtrC is a novel sRNA specific to *Francisella*.

To study the role of FtrC, we constructed a mutant strain harboring a deletion of the *ftrC* gene (LVSΔ*ftrC*) and a strain expressing FtrC at high levels (LVS/p*ftrC*+). This latter strain contains a plasmid expressing FtrC from a highly potent *Francisella* promoter, the Pbfr promoter [26]. Expression and approximate size of FtrC in wild-type LVS was confirmed by Northern blot analysis (Fig. 1C), whereas no transcript was detected in the LVSΔ*ftrC* strain. The Northern blot showed that LVS/p*ftrC*+ expressed FtrC at a high level, and this was further verified by quantitative reverse transcription PCR (qRT-PCR) that demonstrated a 46-fold increase in the FtrC RNA level compared to the LVS strain (data not shown). Both of the constructed strains were able to grow in liquid growth medium similarly to the wild-type LVS strain (Fig. S1 and data not shown), which indicated that neither overexpression, nor deletion of the *ftrC* gene had any effect on bacterial survival in broth.

Many sRNAs in Gram-negative bacteria rely on the RNA chaperone Hfq for stability and/or promoting interaction with their target(s). To examine if the stability of FtrC is increased in the presence of Hfq, we assessed the stability of FtrC in wild-type LVS and Δ*hfq* strains (Fig. 1D). As shown in figure 1D, FtrC is relatively stable in the wild-type strain (half-life of ∼13 min) and this was unchanged in LVSΔ*hfq*, showing that Hfq plays no (or only a very minor) role in stabilization of FtrC.

As sRNAs in other bacterial species have been demonstrated to play roles in adaptation to environmental changes, we tested whether the *ftrC* deletion or overexpressing strains were impaired in survival under stress. However, none of the strains showed any change in their ability to survive oxidative stress (10 mM H_2_O_2_), increased osmolarity (2% NaCl), or membrane stress (0.05% SDS) (Fig. S1 and data not shown). This indicates that FtrC is not involved in controlling expression of genes participating in resistance to either of these stresses.

### FtrC affects expression of target gene

Most sRNAs control the expression of target genes by interacting with their mRNA. The *ftrC* gene is found in the IGR between *FTL_0777* and *FTL_0778* and its sequence does not overlap those of the flanking genes. Although it is possible that FtrC could base-pair with the 5′UTR of gene *FTL_0778* it indicates that its target(s) is located in trans. Base-pairing between a sRNA and target mRNA generally leads to changed translation and often the stability of the mRNA is affected as well. Therefore, to identify possible targets of FtrC, we compared the transcriptomes of the LVSΔ*ftrC* and LVS/p*ftrC*+ strains after growth in liquid broth. This allowed us to identify four genes for which the mRNAs were found to have a different mRNA level when FtrC was overexpressed (table 1). We used qRT-PCR to examine the results obtained by microarray analysis and this verified that the *FTL_1293* transcript was found at a lower level in the LVS/p*ftrC*+ strain (4.3-fold, see table 1). To address if this effect was specific to FtrC, we constructed a plasmid that overexpresses another *F. tularensis* sRNA, the FtrB sRNA [25]. This plasmid expresses FtrB from the same promoter used for FtrC and resulted in more than 100-fold overexpression of FtrB compared to the wild-type strain (data not shown). However, overexpression of FtrB did not affect *FTL_1293* transcript levels (data not shown), indicating that the lower level observed in the LVS/p*ftrC+* strain was due to high FtrC expression.

**Table 1:** Effect of overexpression of FtrC on the transcript level of four genes^a^.

		Fold repression^b^	
Gene	Gene product	Microarray	qRT-PCR
*FTL_0880*	hypothetical protein	0.67	1.1
*FTL_0698*	hypothetical protein	1.5	1.1
*FTL_0881*	hypothetical protein	1.5	1.0
*FTL_1293*	hypothetical protein	2.2	4.3

a Four genes found to have consistently changed mRNA levels in DNA microarray study.

b Fold difference in RNA level in LVSΔ*ftrC* relative to LVS/p*ftrC*+ strain.

We furthermore used the program TargetRNA (http://snowwhite.wellesley.edu/targetRNA/) [27], [28] to predict putative targets for FtrC. Initially the search was focused around the start codon of annotated genes in the LVS genome (where most sRNAs bind to affect ribosome binding) and 20 putative targets were found (table S1). We checked by qRT-PCR whether the 20 putative targets had a changed mRNA level in the LVS/p*ftrC*+ strain compared to the *ftrC* mutant strain, but found no significant difference for any of the genes (data not shown). We therefore decided to perform the TargetRNA search within the coding region of possible target genes and *FTL_1293* came out as the most likely target of 31 putative target genes (table S2). Since we also had identified gene *FTL_1293* in our transcriptomic study and the changed mRNA level was confirmed by qRT-PCR (table 1), we pursued this gene as a likely target for FtrC. *FTL_1293* encodes a putative transcriptional regulatory protein.

### FtrC binds to *FTL_1293* RNA

**Figure 2:**
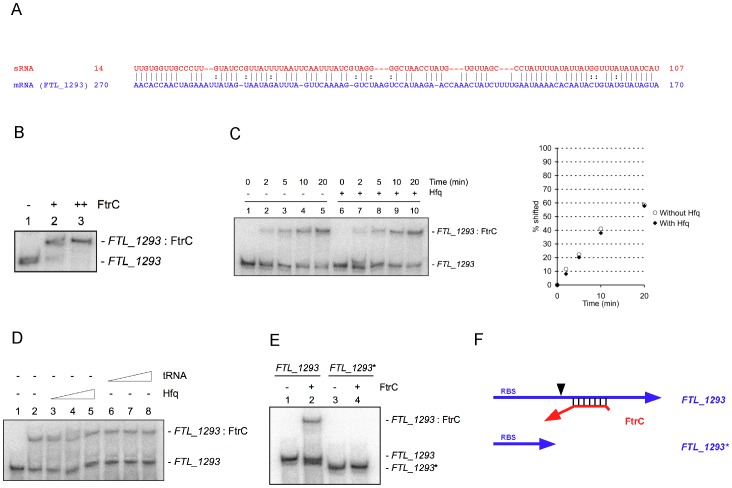
FtrC specifically binds *FTL_1293* mRNA. (A) Putative duplex formation between FtrC and *FTL_1293* mRNA predicted by TargetRNA. The positions of basepairing nucleotides relative to transcript start (for FtrC) or AUG codon (for *FTL_1293*) are indicated at left and right to the sequences. (B) Gel shift assay of ^32^P end-labeled *FTL_1293* mRNA incubated with increasing amounts of FtrC for 60 min before loading on a native 5% acrylamide gel. (C) ^32^P end-labeled *FTL_1293* mRNA was incubated for the indicated time with FtrC before loading and RNA-RNA complex formation assessed by gel shift assay. When indicated, 10 µM Hfq was included during RNA-RNA incubation. Quantification of complex formation over time is presented at right. (D) ^32^P end-labeled *FTL_1293* mRNA incubated with FtrC in the presence of increasing amount of Hfq or tRNA and interaction analyzed by native gel electrophoresis. Lane 1: no FtrC was added to reaction. (E) ^32^P end-labeled full-length (*FTL_1293*) or truncated *FTL_1293* mRNA (FTL_1293*) incubated with or without FtrC and analyzed by native acrylamide gel electrophoresis. (F) Schematic representation of putative interaction between FtrC and *FTL_1293* mRNA (top) and the extent of the truncated mRNA (FTL_1293*) (bottom).

TargetRNA predicted binding of FtrC inside the coding sequence (CDS) of *FTL_1293*, with a possible interaction over a region of ∼100 nt (Fig. 2A). To test whether the two RNA molecules interact *in vitro*, we performed gel shift assays. FtrC and *FTL_1293* mRNA (complete coding sequence and 26 nt upstream the start codon) were produced by *in vitro* transcription and *FTL_1293* RNA was end-labeled with ^32^P. When the RNAs interacted for 60 min before loading onto a native polyacrylamide gel, a retarded band appeared when 10- and 100-fold excess FtrC was added (Fig. 2B). 100% of the RNA was found in the retarded complex with 100-fold excess FtrC (Fig. 2B, lane 3), whereas a small fraction was unbound when 10-fold excess was added (Fig. 2B, lane 2). This demonstrated that a *FTL_1293*-FtrC duplex was formed *in vitro*.

### FtrC binding is Hfq-independent

We next assessed the binding over time. As can be seen in figure 2C, a fraction of *FTL_1293* mRNA was found in the retarded complex after 2 min of interaction and the amount increased with longer times of incubation. Although the Hfq protein did not seem to affect stability of FtrC, we examined if it played any role in promoting RNA-RNA interaction between FtrC and *FTL_1293* mRNA. The formation of the RNA-RNA complex was not affected by addition of Hfq, both the amount of complex and the rate of formation were unchanged in the presence of Hfq (Fig. 2C). Furthermore, increasing the amount of Hfq protein did not affect duplex formation (Fig. 2D, lanes 1–5). This strongly indicates that FtrC is not dependent on Hfq for function or for stability. The interaction between FtrC and *FTL_1293* mRNA was specific, as addition of excess tRNA had no effect on duplex formation (Fig. 2D, lanes 6–8). The predicted interaction between FtrC and *FTL_1293* mRNA involves the central part of the mRNA and not the 5′ region containing the ribosome binding site (RBS) where many bacterial sRNAs bind. We therefore produced a truncated mRNA (FTL_1293*) (see Fig. 2F) where the nucleotides after position +150 (relative to the AUG) were omitted and asked whether FtrC could bind to this RNA. As can be seen in figure 2E, no interaction between FtrC and FTL_1293* mRNA was observed whereas FtrC formed a complex with *FTL_1293* mRNA under the same conditions. This shows that FtrC binds to *FTL_1293* mRNA, but requires determinants in the CDS of the target gene.

### FtrC modulates *F. tularensis* intracellular replication and virulence in mice

**Figure 3:**
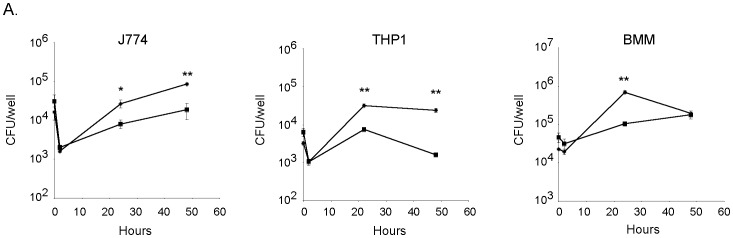
Expression of FtrC reduces intracellular multiplication of *F. tularensis*. Murine macrophage-like cells J774, human THP1 cells, and murine bone marrow-derived macrophages (BMM) were incubated with LVS/p6 (diamonds) or LVS/p*ftrC*+ (squares) bacteria. After 60 min the cells were washed and gentamycin added to kill extracellular bacteria (time 0). The number of intracellular bacteria was determined after lysis of macrophages cells and plating of lysate on agar plates. Results are from one representative experiment (triplicate samples). Student's t-test showed difference in bacterial numbers (* designates p<0.05, and ** designates p<0.005).

An important aspect of *Francisella* virulence is its capacity to replicate inside the cytoplasm of host cells, particularly in macrophages. We therefore assessed if the LVSΔ*ftrC* and LVS/p*ftrC*+ strains were able to infect and multiply inside murine macrophage-like J774 cells. We found that LVSΔ*ftrC* multiplied in a manner indistinguishable from the LVS strain, implying that *ftrC* is dispensable for intracellular replication (Fig. S2). In contrast, the strain producing FtrC at a high level exhibited reduced multiplication, but was nevertheless able to replicate inside J774 cells (Fig. 3). At 24 h post-infection, LVS/p6 had reached 3-fold higher numbers than LVS/p*ftrC*+, and at 48 h the difference was about 5-fold. Similar results were obtained with the human macrophage cell-line THP1 and murine bone marrow-derived macrophages (BMMs) (Fig. 3). In THP1 macrophages, the differences in numbers between the two strains were 4-fold at 24 h post-infection and 15-fold at 48 h, whereas we only observed a difference at 24 h in BMMs (∼7-fold). This showed that increased levels of FtrC diminish the capacity of *F. tularensis* to replicate inside host cells, but does not abolish intracellular multiplication.

**Figure 4:**
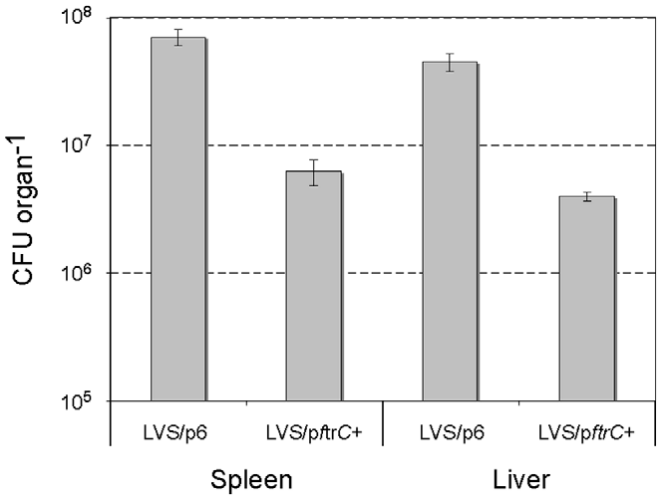
Expression of FtrC reduces the number of bacteria in spleen and liver of infected mice. The numbers of bacteria in the spleen and liver of mice infected with strain LVS/p*ftrC*+ or LVS/p6 were determined after 3 days of infection with approximately 500 bacteria. Student's t-test shows a significant difference between the numbers of bacteria in spleen and liver of mice infected with the two strains (p<0.0005).

To further investigate the role played by FtrC in *Francisella* pathogenesis, we performed *in vivo* virulence studies in the mouse model. Five BALB/c mice were infected with ∼500 bacteria (LVS/p6 or LVS/p*ftrC*+) by the intra-peritoneal (i.p.) route, a dose corresponding to ∼50-fold the LD_50_ of LVS by this route. After 3 days of infection, when bacteria have disseminated and colonized the spleen and liver, the mice were sacrificed and organs removed and the number of bacteria determined. At this time we recovered an average of 7×10^7^ and 5×10^7^ colony forming units (CFU) from the spleen and liver, respectively, from the mice infected with the LVS/p6 strain (Fig. 4). In contrast, in mice infected with the FtrC overexpressing strain, about 6×10^6^ and 4×10^6^ CFU colonized the spleen and liver (Fig. 4). This demonstrated that high expression of FtrC decreased the number of bacteria recovered from both the spleen and liver about 10-fold.

Taken together, these experiments showed that overexpression of FtrC results in decreased replication of *F. tularensis* in macrophages *in vitro* and it lowers the capacity of the bacterium to survive and/or multiply in the organs of infected mice *in vivo*. This indicates that FtrC can function as a regulatory element to modulate the virulence of *F. tularensis*.

### The FtrC target FTL_1293 is not required for *F. tularensis* virulence

**Figure 5:**
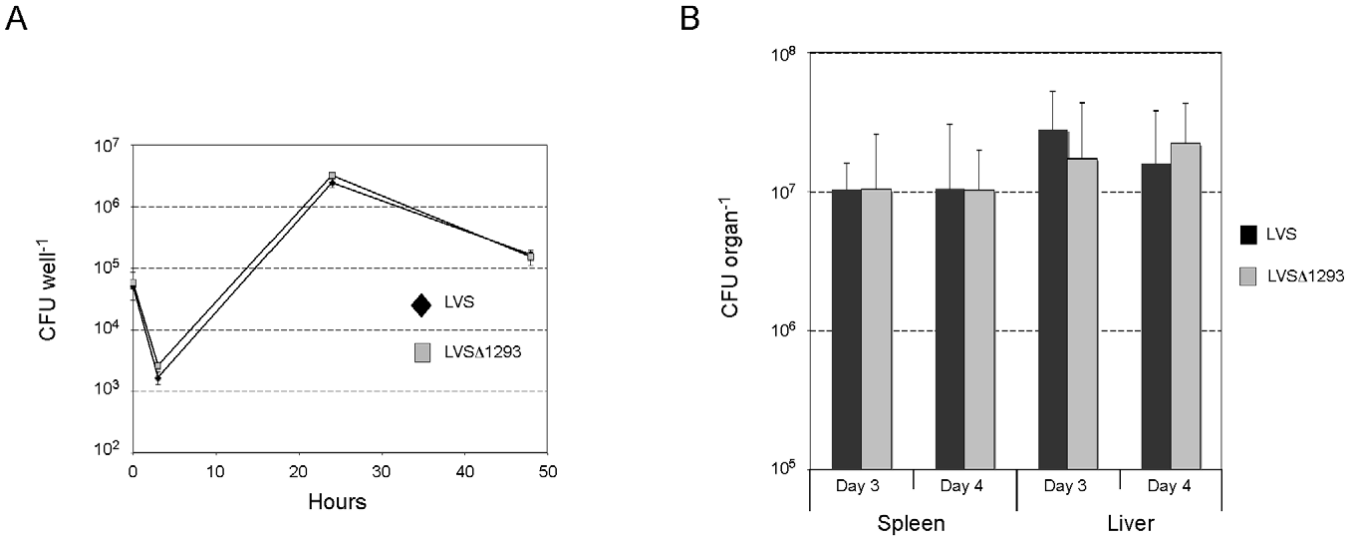
FTL_1293 does not contribute to *F. tularensis* virulence. (A) Intracellular multiplication of LVS (black diamonds) and LVSΔ1293 (grey squares) in murine macrophages-like J774 cells. After 60 min the cells were washed and gentamycin added to kill extracellular bacteria (time 0). The number of intracellular bacteria was determined after lysis of macrophages cells and plating of lysate on agar plates. Results are from one experiment with triplicate samples. (B) Average viable numbers of LVS (black bars) and LVSΔ1293 (grey bars) in the spleen and liver of five mice after 3 and 4 days of infection by the i.p. route with approximately 100 bacteria.

As high expression of FtrC reduces intracellular replication of *Francisella* it raises the question whether this is a result of the lower expression of FTL_1293 observed when FtrC is overexpressed. To address this, we created a mutant strain with a deletion of gene *FTL_1293* (LVSΔ1293) and tested this strain for its ability to replicate in macrophages *in vitro*. However, we found that LVSΔ1293 replicates in murine macrophages-like J774 cells in a manner indistinguishable from the wild-type strain (Fig. 5A). Even though FtrC was not necessary for replication in macrophages *in vitro*, we next assessed whether it contributed to virulence in a mouse model. We infected mice with approximately 100 bacteria, wild-type or mutant, and determined the number of bacteria in the spleen and liver after 3 and 4 days of infection. This resulted in the recovery of the same numbers of bacteria from both liver and spleen of mice infected with either the LVS or LVSΔ1293 strains (Fig. 5B). Taken together, these results demonstrate that gene *FTL_1293* plays no role in the intracellular replication of *Francisella in vitro* and *in vivo*. Hence, the negative effect of high FtrC expression on bacterial virulence cannot be explained by its role in control of *FTL_1293* expression. It therefore seems likely that other FtrC target(s) remains to be identified.

## Discussion

In this study, we identify and characterize for the first time a sRNA, FtrC, that modulates the virulence capacities of *Francisella tularensis*. We show that overproduction of this ∼200 nt sRNA impairs both intracellular multiplication and virulence. We also identify a target of FtrC and the region of interaction between FtrC and the target mRNA.

### *ftrC* is conserved and affects bacterial replication in macrophages

*ftrC* is found in an intergenic region (IGR) where most genes encoding bacterial *trans*-acting sRNAs are located. A *ftrC* gene is found in different strains of all subspecies (subsp.) of *F. tularensis* showing 98–100% sequence identity. Notably, *F. tularensis* subsp. *novicida* strain U112 contains two tandem copies of *ftrC* (90 and 98% identity to the LVS gene) and two additional, but shorter copies (with 85 and 97% identify) at other locations. A copy of *ftrC* is also found in *F. philomiragia*, but the gene is shorter than the LVS counterpart. Strikingly, we could not find any *ftrC* homologues in other bacterial species by BLAST searches and the Rfam database did not contain any RNA families with homology to FtrC. It therefore seems likely that FtrC is a *Francisella*-specific sRNA.

**Figure 6:**
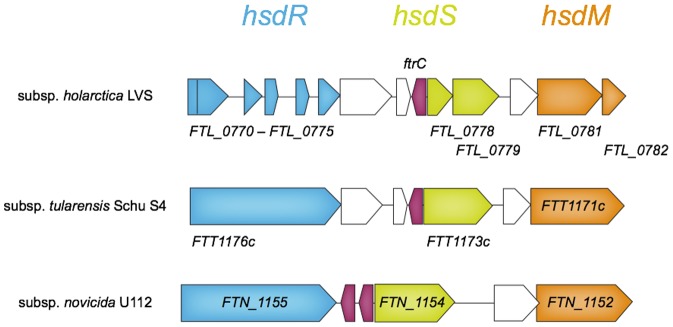
The type I RM system locus containing *ftrC*. In *F. tularensis* subsp. *novicida* U112, the restriction subunit R (HsdR) is encoded by *FTN_1155* (blue), the specificity subunit S (HsdS) is encoded by *FTN_1154* (green) and the modification subunit M (HsdM) is encoded by *FTN_1152* (orange). The corresponding pseudogenes (indicated with gene names below arrows) in *F. tularensis* subsp. *holarctica* LVS and subsp. *tularensis* Schu S4 are represented using the same colors. The *ftrC* gene (red) is encoded on the opposite strand.

The region harboring *ftrC* seems to have undergone extensive genomic changes. In *F. tularensis* subsp. *novicida*, the region containing the two successive *ftrC* genes encodes a functional type I restriction-modification system [29], whereas the *ftrC* regions in subspecies *holarctica* and *tularensis* contain remnants of a restriction-modification system (Fig. 6). Even though the surrounding genes are non-functional, both of these subspecies have retained one copy of the *ftrC* gene, suggesting that the function of this gene is not dispensable.

FtrC is not necessary for growth *in vitro* in broth and in macrophages, as a *ftrC* mutant multiplies in liquid broth and in macrophages similarly to the wild type LVS strain. However, high expression of FtrC impairs bacterial multiplication in macrophages *in vitro* and reduces the number of bacteria in the organs of infected mice. This effect is not due to a general decreased capacity of the bacteria to multiply, as we observed no difference in growth in liquid broth. Therefore, it is probable that FtrC regulates expression of a gene (or several genes) that is important for the intracellular replication of *F. tularensis*.

### FtrC interacts specifically with target mRNA

We successfully identified a target for FtrC regulation by combining DNA microarray based transcriptional profiling (and qRT-PCR) with *in silico* target prediction. FtrC negatively regulates expression of the target, *FTL_1293*, since overexpression of FtrC results in decreased amount of *FTL_1293* mRNA. *FTL_1293* encodes a relatively short (156 amino acids) protein that is 100% conserved among the *F. tularensis* subspecies. In the N-terminal region, the protein contains a helix-turn-helix domain found in the ArsR family of bacterial transcriptional regulators, suggesting that it might function as a transcriptional regulatory protein in *F. tularensis*. If so, regulation by FtrC is a two-step process in which FtrC-based regulation of FTL_1293 indirectly affects transcription of downstream genes.

Notably, the *F. tularensis* subsp. *novicida* homologue (*FTN_1534*) of *FTL_1293* was identified in two different genome-wide screens that searched for genes involved in bacterial survival and/or replication in mice and for replication in *Drosophila* macrophage-like S2 cells [30], [31], respectively. Also, a transcriptional study in *F. tularensis* subsp. *tularensis* has demonstrated that *FTL_1293* transcript levels change during the intracellular cycle [15], further indicating a role for this gene for intracellular growth and/or survival. However, our data demonstrate that *FTL_1293* in strain LVS does not contribute to intracellular multiplication in murine macrophages or in organs of infected mice. This indicates that FtrC controls more than one target gene and that the regulation of this other(s) target(s) is responsible for the decrease in intracellular multiplication we observe when FtrC is expressed at high levels.

Many bacterial *trans-*acting sRNAs rely on the RNA chaperone Hfq for function and stability. Our data show that FtrC stability is unchanged in a *hfq* mutant and that the interaction between FtrC and the *FTL_1293* transcript is not affected by Hfq. Hence FtrC appears to be Hfq-independent.

Bacterial sRNAs generally regulate expression of target genes by base-pairing with their target mRNA in the 5′ region, thereby preventing ribosome binding and often simultaneously promoting RNA degradation. Our data show that FtrC binds to a full-length *FTL_1293* mRNA, but not to a RNA consisting of only the 5′ region and the initial 150 nt of the coding sequence (CDS). This strongly suggests that FtrC binds to a sequence within the CDS of *FTL_1293*, although downstream coding sequences could be required for proper folding of the RNA and not for duplex formation *per se*. Some bacterial sRNAs have been reported to bind within the CDS and nevertheless control ribosome binding, but it has been suggested that only interactions down to the fifth codon are likely to repress translational initiation [32]. This finding and the location of the supposed interaction between FtrC and *FTL_1293* mRNA suggest that FtrC acts not by sterically hindering ribosome binding, but asserts its regulatory effect on FTL_1293 expression by another mechanism. The SR1 RNA of *Bacillus subtilis* binds its target mRNA around position +80 relative to the AUG and induces a structural change that inhibits ribosome binding [33]. In *Salmonella typhimurium*, the sRNA MicC regulates expression of *ompD* by forming a short RNA duplex within the CDS [34]. This duplex does not affect translational initiation but instead accelerates mRNA degradation.

We have identified a novel sRNA in *F. tularensis* LVS that reduces intracellular replication when expressed at high level. This sRNA, FtrC, binds to the mRNA of a target gene, *FTL_1293*, and affects its mRNA level. Since we observe that *FTL_1293* does not play any role in *F. tularensis* intracellular multiplication *in vitro* or *in vivo*, it seems probable that other target(s) of FtrC involved in modulating virulence remains to be identified.

## Materials and Methods

### Ethics Statement

All animal experiments were carried out in accordance to the European guidelines and following the recommendations of the INSERM guidelines for laboratory animals' husbandry. The protocol was approved by the INSERM Ethics Committee (Authorization Number: 75-906).

### Bacterial growth conditions

All bacterial strains, plasmids and primers used in this study are described in Table S3.

*F. tularensis* LVS and derivatives were grown in Schaedler broth supplemented with vitamin K3 (BioMerieux, France), in a chemically defined medium (CDM) [35], or on chocolate agar plates (BioMerieux) at 37°C. When needed, media were supplemented with 10 µg ml^−1^ kanamycin. All DNA manipulations were done in *E. coli* TOP10 and *E. coli* strains were grown in LB broth.

### cDNA cloning

Size fractionation, RNA elution and cDNA cloning were done as described [25]. Briefly, 20 µg of total RNA was loaded on an 8% polyacrylamide/8 M urea gel. After separation RNA was eluted from the gel after crushing and incubation at 37°C in 0.5 M ammonium acetate and 1 mM EDTA (pH 8.0). Supernatants were extracted once with chloroform before precipitation of the RNA with isopropanol in the presence of glycogen. Next, 3′ adapter was ligated to the dissolved RNA with T4 RNA ligase and the RNA was purified from a 15% polyacrylamide/urea gel to remove non-incorporated adaptors. The eluted RNA was treated with tobacco acid phosphatase (TAP) before ligation of 5′ adapter and subsequently non-incorporated adapters removed by passing and twice washing the RNA on a Microcon YM-30 column (Millipore). The RNA was reverse transcribed using the 3′PRIMER and SuperScript II reverse transcriptase (Invitrogen), PCR amplified using 5′PRIMER and 3′PRIMER and ligated into pCR2.1-TOPO (Invitrogen).

### Strain construction

The *ftrC* deletion mutant was created by allelic exchange using the *sacB*-based suicide vector pMP812 [36]. Regions of approximately 1 kb upstream and downstream of *ftrC* were amplified by PCR using primer pairs DelR/DelB and DelO/DelT. The upstream and downstream fragments were purified from gel, annealed and extended in 20 cycles of PCR without primers and the product further used as template in a PCR reaction with primers DelR and DelT. The ∼2 kb PCR products were cloned into pCR2.1-TOPO and mobilized as a *Not*I-*Sal*I fragment into vector pMP812 creating pMP-Δ*ftrC*. pMP-Δ*ftrC* was introduced in LVS by electroporation and integration of the plasmid into the chromosome confirmed by PCR. Strains were then passed once in medium without selection, subsequently streaked on solid medium containing 5% sucrose and isolated colonies were tested for loss of the gene by PCR (using primer pairs DelCheck1/DelCheck4 and DelCheck2/DelCheck3). Deletion of the gene was confirmed by sequencing.

The plasmid expressing FtrC at high level, p*ftrC*+, was created by amplifying the Pbfr promoter [26] (using primers Pbfr_F and Pbrf_R) and the *ftrC* gene (using primers FtrC_ovx_F and FtrC_ovx_R) from the LVS genome. The two PCR products were cloned into pCR2.1-TOPO and then subcloned into the *Nde*I and *Nhe*I sites of pFNLTP6 [37]. The plasmid was introduced in *F. tularensis* LVS by electroporation.

The plasmid expressing FtrB at high level, p*ftrC*+, was created by amplifying the Pbfr promoter [26] (using primers Pbfr_FE and Pbrf_R_FtrB) and the *ftrB* gene (using primers FtrB_ovx_F and FtrB_ovx_R) from the LVS genome. A sawing PCR product (using primers Pbfr_FE and FtrB_ovx_R) was then cloned into the *EcoR*I of pFNLTP6 [37] and sequenced. The plasmid was introduced in *F. tularensis* LVS by electroporation.

The *FTL_1293* deletion mutant was constructed essentially as described for the *ftrC* mutant. The initial PCRs were performed with primer pairs 1293_AF/1293_AR and 1293_BF/1293_BR and the ∼2 kb PCR fragment produced by sawing PCR (using primers AF and BR) was cloned into pMP812 as a *Sal*I-*Bam*HI fragment. The complete sequence of the plasmid insert was verified and loss of the wild-type gene in the mutant strain confirmed by PCR (using primers 1293_up and 1293_down).

### Northern blot

Total RNA was extracted using TRIzol according to the manufacturer's instructions (Invitrogen) and quantified on a NanoDrop ND-1000 Spectrophotometer (Thermo Scientific). After extraction, 10 µg of total RNA were loaded on a polyacrylamide gel (8% acrylamide 19∶1, 8 M urea). After migration, the RNA was transferred to a Hybond-N+membrane (Amersham) and crosslinked with UV light. The membrane was prehybridized in Rapid-Hyb Buffer (Amersham). Then, ^32^P-labelled gene-specific probe (oligonucleotide, see table S3) was added directly in the prehybridization buffer with the membrane and incubated for 16 h at 42°C. After hybridization, the membrane was washed twice with 2×SSC/0.1% SDS, once with 1×SSC/0.1% SDS and twice with 0.1×SSC/0.1% SDS. Results were analyzed on a Storm 860 PhosphorImager (Molecular Dynamics) using the ImageQuant software (Molecular Dynamics).

For stability experiments, rifampicin (500 µg ml^−1^) was added to bacterial cultures at OD_600 nm_ = 0.4 and RNA was isolated after 0, 2, 5, 10, 20, and 30 min.

### 5′- and 3′- RACE

For 5′-RACE, 15 µg of total RNA was incubated with TAP for 2 hours at 37°C after which 5′ adapter was ligated with RNA ligase. RNA was purified from a gel and reverse transcribed with a gene specific primer and SuperScript II RT. cDNA was then used as a template in PCR reaction with the 5′PRIMER and the gene specific primer (GSP_5′RACE) and cloned into pCR2.1-TOPO before sequencing. Seven individual clones were sequenced.

3′-RACE was performed by ligating the 3′ adapter to total RNA, followed by gel purification of adaptor ligated RNA, reverse transcription with 3′PRIMER and finally conducting PCR with 3′PRIMER and GSP_3′RACE. The 3′ end of each sRNA was determined by sequencing PCR products cloned in pCR2.1-TOPO. Ten clones were sequenced.

### Quantitative reverse transcription PCR

One µg RNA was reverse transcribed using random hexamers and SuperScript II reverse transcriptase (Invitrogen) according to the protocol provided by the manufacturer. Real-time PCR was performed with gene-specific primers (table S3) using an ABI PRISM 7700 and SYBR green PCR master mix (Applied Biosystems, Foster City, CA). To calculate the amount of transcript, a standard curve was plotted for each gene-specific primer set using a series of diluted genomic DNA from LVS. To compare the transcript levels of LVS (or LVSΔ*ftrC*) with that of LVS/p*ftrC*+, the amounts of transcript was normalized to DNA helicase (*FTL_1656*), as this gene has been shown to change little in expression during growth [8]. The fold difference between the two strains was calculated from triplicate samples.

### Cell infection

J774 cells (ATCC, number TIB-67) were propagated in Dulbecco's Modified Eagle's Medium (DMEM) or RPMI medium containing 10% fetal calf serum. Cells were seeded at a concentration of ∼2×10^5^ cells per well in 12-well tissue plates (Falcon) and monolayers were used 24 hours after seeding. J774 macrophage monolayers were incubated for 1 hour at 37°C with the bacterial suspensions (approximate multiplicity of infection 100) to allow the bacteria to enter.

THP1 cells (ATCC, number TIB-202) were grown in RPMI containing 10% fetal calf serum. Approximately 2×10^5^ cells/well were dispensed in 24-well tissue culture plates (Falcon) and treated for 48 hours with 200 ng ml^−1^ phorbol myristate acetate to make the cells adherent. Bacteria (approximate multiplicity of infection of 50) were allowed to infect THP1 cells for 1 hour.

Bone marrow-derived macrophages (BMM) from BALB/c mice were obtained and cultured as described [38]. Cell monolayers were incubated for 1 hour at 37°C with the bacterial suspensions in DMEM (average multiplicity of infection of 200) to allow the bacteria to enter.

After washing (time zero of the kinetic analysis), the cells were incubated in fresh culture medium containing gentamicin (10 µg ml^−1^) to kill extracellular bacteria. At several time-points, cells were washed three times in PBS and processed for counting of surviving intracellular bacteria. For this, bacteria were recovered by lysis of macrophages with distilled water and the titer of viable bacteria released from the cells was determined by spreading preparations on agar plates. For each strain and time in an experiment, the assay was performed in triplicate. Each experiment was independently repeated two times (for FtrC overexpreesion) and the data presented are from one experiment. The experiment with LVSΔ1293 was performed once with triplicate samples for each time point and with an approximate multiplicity of infection of 150.

### Mice virulence assay

All animal experiments were carried out in accordance to the European guidelines and following the recommendations of the INSERM guidelines for laboratory animals' husbandry.

LVS/p6 and LVS/p*ftrC*+ were grown in Schaedler-K3 containing kanamycin to exponential growth phase and diluted to the appropriate concentration. 6–8 weeks old female BALB/c mice (Janvier, Le Genest-St-Isle, France) were injected each day subcutaneously with kanamycin (50 µl of 12 mg/ml solution) for three days before and during the infection. Mice were i.p. inoculated with 200 µl of bacterial suspension (corresponding to approximately 500 CFU). The actual number of viable bacteria in the inoculum was determined by plating dilutions of the bacterial suspension on chocolate plates. After four days, the mice were sacrificed. Homogenized spleen and liver tissue from the five mice were diluted and spread onto chocolate agar plates supplemented with kanamycin and the number of viable bacteria per organ determined.

For experiment with LVSΔ1293, mutant and wild-type strains were grown to exponential phase and diluted to the appropriate concentration. Five mice were i.p. inoculated with 200 µl of bacterial suspension (corresponding to approximately 100 CFU) of either strain. After 3 and 4 days of infection, the mice were sacrified and homogenized liver and spleen tissues were diluted and spread onto chocolate agar plates to determine the number of viable bacteria.

### cDNA labeling and microarray hybridizations

RNA used in microarray experiments was extracted using TRIzol reagent combined with purification of the aqueous phase on RNeasy columns (Qiagen). cDNA was labeled and microarray hybridizations performed as described [24]. Two independent experiments and RNA extractions were performed and each set of RNAs was used in one hybridization experiment. The *F. tularensis* microarrays (obtained from the “pathogen functional genomics resource center”, PFGRC) contain 70-mer oligonucleotides representing all genes of strains SchuS4 and LVS in five copies. Microarrays were scanned with a Genepix 4000B scanner (Molecular Devices). To quantify signal fluorescence intensities, TIFF images were analyzed using the Genepix Pro 6.0 software. Statistical analyses were performed using publicly available software, the R/Bioconductor package LIMMA (available from www.bioconductor.org). A list of statistically significant differentially expressed genes was obtained using lowess normalization (after inspection of MA plots) and applying the empirical Bayes moderated t-test. Data has been submitted to the ArrayExpress database (accession number: E-MEXP-3424).

### Hfq purification

Hfq_FTU_ was purified using the Intein system (Impact-CN; New England Biolabs) basically as described [39]. Briefly, the *F. tularensis hfq* gene was amplified using primers Hfq-IMPACT-Up and Hfq-IMPACT-Down and cloned as a *Sap*I/*Eco*RI fragment into plasmid pTYB21 and the resulting plasmid pTYB21-Hfq_FTU_ was sequenced. Hfq_FTU_ was expressed in *E. coli* strain ER2566 by adding 0.4 mM IPTG for 16 hrs at 15°C. The recombinant protein was purified according to the protocol provided by the manufacturer.

### In vitro transcription

Templates for *in vitro* transcription of FtrC, *FTL_1293* and FTL_1293* were constructed by PCR using the primers listed in Table S3. The templates contain a 5′-end T7 promoter. *In vitro* transcription was performed using the MegaScript kit as described by the manufacturer (Ambion). *In vitro* transcribed RNA was ethanol precipitated, resuspended in formamide loading dye and separated on an 8% denaturing polyacrylamide gel. The RNA was visualized by UV shadowing, excised from the gel and transferred to 600 µl 0.5 M NH_4_-acetate containing 1 mM EDTA. After 6 h incubation at 37°C, the RNA was phenol extracted followed by isopropanol precipitation. Quantification was performed on a NanoDrop 2000. *In vitro* transcribed RNA was 5′-end-labelled using the T4 Polynucleotide Kinase kit as described by the manufacturer (Fermentas).

### Gel shift assay

For gel shifts, 10 fmol 5′-end-labelled *FTL_1293* or FTL_1293* RNA was incubated in a total of 10 µl with or without 10 nM unlabelled FtrC in the absence or presence of 0.1, 0.5 or 1 µM Hfq_FTU_ and 0.25, 2.5 or 5 µg tRNA. The samples were incubated 20 min at 37°C followed by 10 min on ice and subsequently separated on a 5% non-denaturing polyacrylamide gel. For time course experiments, 10 fmol 5′-end-labelled *FTL_1293* RNA was mixed with 10 nM FtrC in the presence or absence of 10 µM Hfq_FTU_ and incubated at 37°C for 2, 5, 10 or 20 min followed by 30 s on ice. The samples were then loaded onto 5% non-denaturing polyacrylamide gel with the current running.

## Supporting Information

Figure S1 **Growth characteristics and stress resistance of the LVS/p** ***trC1*** **+ and LVS/p6 strains.** (A) Growth of LVS/p*ftrC*+ and LVS/p6 strains in Schaedler medium containing vitamin K3. (B) Growth of LVS/p*ftrC*+ and LVS/p6 strains in Chamberlain defined medium. Data shown are from experiments with the strain over-expression FtrC, but similar results were obtained with the LVSΔ*ftrC* strain (not shown). For stress resistance assays, exponential-phase bacteria were diluted to a final concentration of 10^8^ bacteria ml^−1^ in fresh Schaedler-K3 broth and subjected to 0.05% SDS (C) and oxidative stress (10 mM H_2_O_2_) (D). The bacteria were plated on chocolate agar plates at different times, and viable bacteria were determined by counting colonies 3 days later. Data are the average CFU ml^−1^ for two independent experiments for each condition. (E) Growth of LVS/p*ftrC*+ and LVS/p6 strains in Schaedler-K3 broth supplemented with 0% or 2% NaCl.
(TIFF)

Figure S2 **Intracellular multiplication of LVS and LVSΔ** ***ftrC*** **in murine macrophage-like J774 cells.** Murine macrophage-like cells J774 were incubated with LVS (diamonds) or LVSΔ*ftrC* (squares) bacteria. After 60 min the cells were washed and gentamycin added to kill extracellular bacteria (time 0). The number of intracellular bacteria was determined after lysis of macrophages cells. Results are from one representative experiment (with triplicate samples).
(TIFF)

Table S1 **Putative targets of FtrC in region of genes surrounding translational start site identified by TargetRNA.**
(DOCX)

Table S2 **Putative targets of FtrC in coding region of genes identified by TargetRNA.**
(DOCX)

Table S3 **Bacterial strains, plasmids and primers used in this study.**
(DOCX)
