# Supplementary figures and images for: Identification of a Novel Small RNA Modulating Francisella tularensis Pathogenicity
Source: PLoS One. 2012 Jul 25;7(7):e41999. doi: 10.1371/journal.pone.0041999 (PMC3405028; doi:10.1371/journal.pone.0041999)

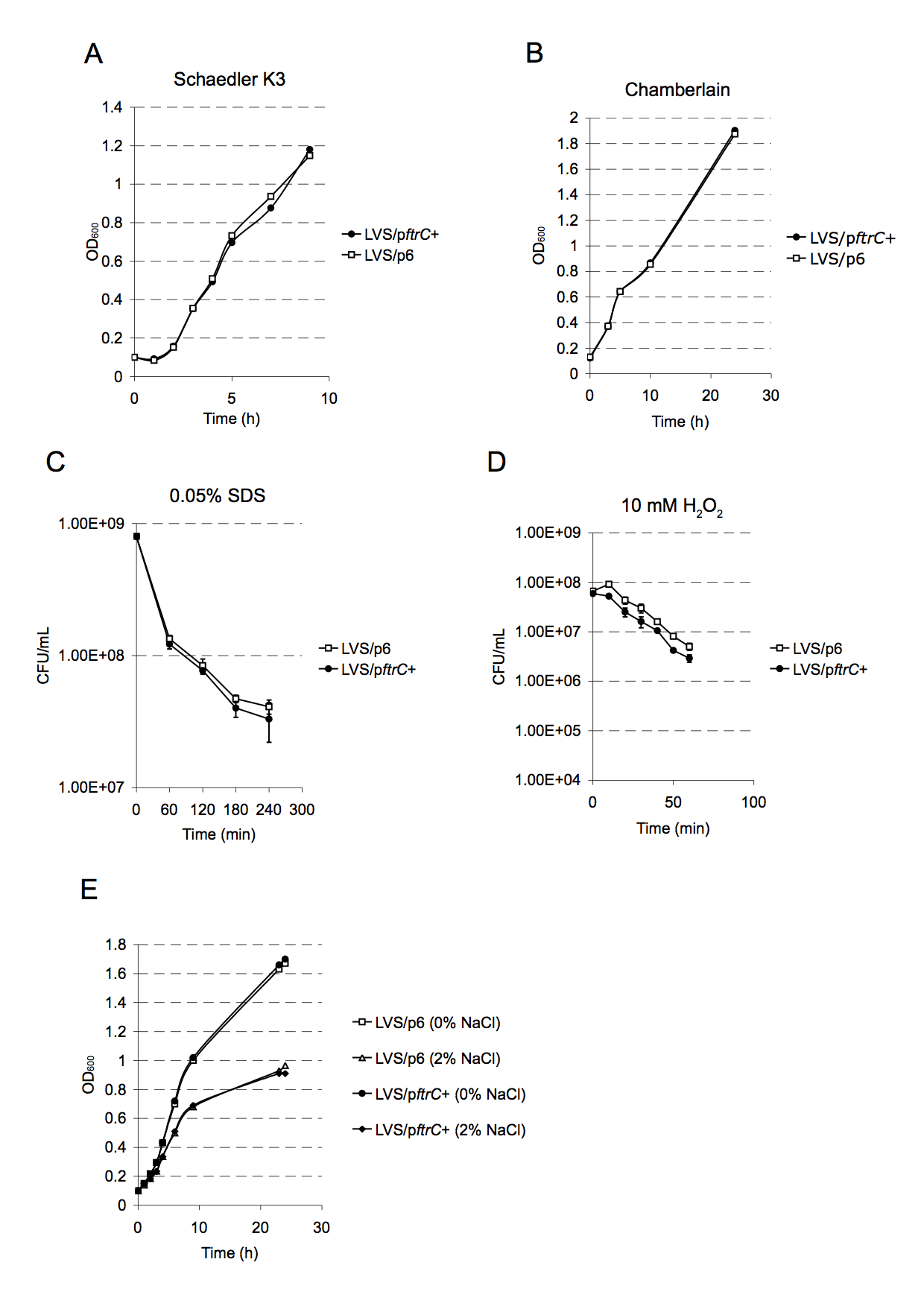

Supplement: Figure S1 — Growth characteristics and stress resistance of the LVS/p trC1 + and LVS/p6 strains. (A) Growth of LVS/pftrC+ and LVS/p6 strains in Schaedler medium containing vitamin K3. (B) Growth of LVS/pftrC+ and LVS/p6 strains in Chamberlain defined medium. Data shown are from experiments with the strain over-expression FtrC, but similar results were obtained with the LVSΔftrC strain (not shown). For stress resistance assays, exponential-phase bacteria were diluted to a final concentration of 108 bacteria ml−1 in fresh Schaedler-K3 broth and subjected to 0.05% SDS (C) and oxidative stress (10 mM H2O2) (D). The bacteria were plated on chocolate agar plates at different times, and viable bacteria were determined by counting colonies 3 days later. Data are the average CFU ml−1 for two independent experiments for each condition. (E) Growth of LVS/pftrC+ and LVS/p6 strains in Schaedler-K3 broth supplemented with 0% or 2% NaCl. (TIFF) [file pone.0041999.s001.tiff]

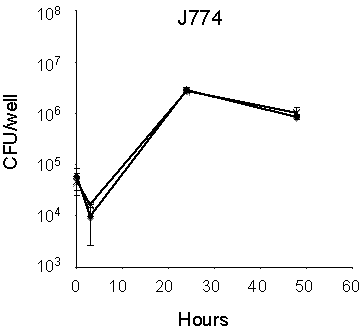

Supplement: Figure S2 — Intracellular multiplication of LVS and LVSΔ ftrC in murine macrophage-like J774 cells. Murine macrophage-like cells J774 were incubated with LVS (diamonds) or LVSΔftrC (squares) bacteria. After 60 min the cells were washed and gentamycin added to kill extracellular bacteria (time 0). The number of intracellular bacteria was determined after lysis of macrophages cells. Results are from one representative experiment (with triplicate samples). (TIFF) [file pone.0041999.s002.tiff]
