# Supplementary material for: Identification of a Novel Small RNA Modulating Francisella tularensis Pathogenicity
Source: PLoS One. 2012 Jul 25;7(7):e41999. doi: 10.1371/journal.pone.0041999 (PMC3405028; doi:10.1371/journal.pone.0041999)
Supplement: Table S1 — Putative targets of FtrC in region of genes surrounding translational start site identified by TargetRNA. (DOCX) [file pone.0041999.s003.docx]

**Table S1. Putative targets of FtrC in region of genes surrounding translational start site identified by TargetRNA**

| Rank | Gene | Synonym | Score | Pvalue | sRNA_start^a^ | sRNA_stop^b^ | mRNA_start^a^ | mRNA_stop^b^ |
| --- | --- | --- | --- | --- | --- | --- | --- | --- |
| 1 | FTL_0447 |  | -78 | 0.000995477 | 82 | 109 | -9 | 20 |
| 2 | FTL_0105 |  | -77 | 0.00117589 | 74 | 94 | -27 | -7 |
| 3 | FTL_1603 |  | -76 | 0.00138898 | 14 | 53 | -19 | 18 |
| 4 | FTL_0754 | *xseA* | -74 | 0.00193789 | 83 | 106 | -19 | 6 |
| 5 | FTL_0548 |  | -71 | 0.00319287 | 83 | 104 | -19 | 6 |
| 6 | FTL_0485 |  | -70 | 0.00377078 | 89 | 109 | -7 | 15 |
| 7 | FTL_1811 |  | -70 | 0.00377078 | 128 | 168 | -21 | 16 |
| 8 | FTL_1806 |  | -69 | 0.00445305 | 84 | 109 | -2 | 20 |
| 9 | FTL_1240 |  | -68 | 0.00525845 | 82 | 104 | -29 | -3 |
| 10 | FTL_0414 | *engA* | -67 | 0.00620906 | 87 | 104 | -30 | -13 |
| 11 | FTL_0857 |  | -67 | 0.00620906 | 35 | 74 | -28 | 4 |
| 12 | FTL_1003 |  | -67 | 0.00620906 | 92 | 110 | -3 | 16 |
| 13 | FTL_0265 |  | -66 | 0.00733088 | 82 | 103 | -20 | 2 |
| 14 | FTL_0633 |  | -66 | 0.00733088 | 25 | 52 | -28 | -1 |
| 15 | FTL_1317 |  | -66 | 0.00733088 | 82 | 103 | -20 | 2 |
| 16 | FTL_1717 |  | -66 | 0.00733088 | 41 | 58 | -21 | -3 |
| 17 | FTL_1889 |  | -66 | 0.00733088 | 68 | 104 | -27 | 13 |
| 18 | FTL_1892 |  | -66 | 0.00733088 | 82 | 103 | -20 | 2 |
| 19 | FTL_0738 |  | -65 | 0.0086545 | 68 | 89 | -21 | 1 |
| 20 | FTL_1210 |  | -65 | 0.0086545 | 81 | 104 | -30 | -3 |

^a^ The first (5’) nucleotide predicted to form RNA-RNA duplex

^b^ The last (3’) nucleotide predicted to form RNA-RNA duplex
