# Supplementary material for: Identification of a Novel Small RNA Modulating Francisella tularensis Pathogenicity
Source: PLoS One. 2012 Jul 25;7(7):e41999. doi: 10.1371/journal.pone.0041999 (PMC3405028; doi:10.1371/journal.pone.0041999)
Supplement: Table S2 — Putative targets of FtrC in coding region of genes identified by TargetRNA. (DOCX) [file pone.0041999.s004.docx]

**Table S2. Putative targets of FtrC in coding region of genes identified by TargetRNA**

| Rank | Gene | Synonym | Score | Pvalue | sRNA_start^a^ | sRNA_stop^b^ | mRNA_start^a^ | mRNA_stop^b^ |
| --- | --- | --- | --- | --- | --- | --- | --- | --- |
| 1 | FTL_1293 |  | -125 | 2.48122e-05 | 14 | 107 | 170 | 270 |
| 2 | FTL_0160 |  | -105 | 0.000498313 | 6 | 107 | 360 | 464 |
| 3 | FTL_1374 |  | -111 | 0.000788291 | 28 | 114 | 1069 | 1148 |
| 4 | FTL_0486 | *glgA* | -109 | 0.000797018 | 6 | 153 | 573 | 721 |
| 5 | FTL_1464 |  | -111 | 0.00084227 | 30 | 106 | 1842 | 1920 |
| 6 | FTL_1468 |  | -111 | 0.00084227 | 30 | 106 | 1842 | 1920 |
| 7 | FTL_1913 |  | -99 | 0.000941236 | 47 | 167 | 349 | 465 |
| 8 | FTL_1066 |  | -99 | 0.00104201 | 7 | 109 | 189 | 291 |
| 9 | FTL_1597 |  | -111 | 0.00113194 | 28 | 114 | 1846 | 1925 |
| 10 | FTL_1338 |  | -103 | 0.00122164 | 13 | 115 | 615 | 713 |
| 11 | FTL_1702 |  | -98 | 0.00128744 | 8 | 93 | 365 | 444 |
| 12 | FTL_0891 | *tig* | -103 | 0.00147556 | 18 | 168 | 162 | 302 |
| 13 | FTL_1332 |  | -90 | 0.00196124 | 82 | 165 | 226 | 309 |
| 14 | FTL_0284 |  | -87 | 0.00295574 | 35 | 104 | 192 | 259 |
| 15 | FTL_1391 | *gmk* | -89 | 0.00371244 | 26 | 83 | 350 | 411 |
| 16 | FTL_1776 |  | -87 | 0.00380204 | 19 | 51 | 155 | 188 |
| 17 | FTL_0516 |  | -91 | 0.0038282 | 43 | 106 | 41 | 102 |
| 18 | FTL_0576 |  | -88 | 0.00382836 | 35 | 110 | 389 | 475 |
| 19 | FTL_0085 |  | -93 | 0.00442052 | 12 | 119 | 311 | 422 |
| 20 | FTL_0835 |  | -89 | 0.0047735 | 82 | 120 | 488 | 529 |
| 21 | FTL_1410 | *murG* | -92 | 0.00485501 | 61 | 113 | 355 | 410 |
| 22 | FTL_1271 |  | -93 | 0.00490258 | 7 | 102 | 471 | 566 |
| 23 | FTL_1213 |  | -95 | 0.00547517 | 42 | 120 | 1613 | 1702 |
| 24 | FTL_1912 | *rpsA* | -94 | 0.00551027 | 1 | 163 | 358 | 508 |
| 25 | FTL_0661 |  | -93 | 0.00609196 | 48 | 100 | 490 | 546 |
| 26 | FTL_1400 |  | -79 | 0.00611872 | 41 | 167 | 58 | 167 |
| 27 | FTL_0762 |  | -83 | 0.00619142 | 145 | 165 | 156 | 176 |
| 28 | FTL_0272 |  | -90 | 0.00649181 | 41 | 92 | 549 | 602 |
| 29 | FTL_1784 | *sucA* | -96 | 0.00685746 | 1 | 58 | 64 | 127 |
| 30 | FTL_1093 |  | -87 | 0.00707904 | 84 | 125 | 167 | 212 |
| 31 | FTL_0731 |  | -85 | 0.00772474 | 8 | 101 | 487 | 578 |
| 32 | FTL_1428 |  | -91 | 0.00793901 | 49 | 109 | 1600 | 1649 |
| 33 | FTL_1142 |  | -87 | 0.00851139 | 15 | 93 | 611 | 688 |
| 34 | FTL_1054 |  | -80 | 0.00944034 | 83 | 109 | 58 | 87 |

^a^ The first (5’) nucleotide predicted to form RNA-RNA duplex

^b^ The last (3’) nucleotide predicted to form RNA-RNA duplex
