# Supplementary material for: Identification of a Novel Small RNA Modulating Francisella tularensis Pathogenicity
Source: PLoS One. 2012 Jul 25;7(7):e41999. doi: 10.1371/journal.pone.0041999 (PMC3405028; doi:10.1371/journal.pone.0041999)
Supplement: Table S3 — Bacterial strains, plasmids and primers used in this study. (DOCX) [file pone.0041999.s005.docx]

Table S3. Bacterial strains, plasmids and primers used in this study.

| **Strain, plasmid or primer** | | | **Description or sequence^a^ (5’->3’)** | **Source, reference or use** |
| --- | --- | --- | --- | --- |
| **Strain** | |  |  |  |
| *F. tularensis* | | |  |  |
|  | LVS | | Subsp. *holarctica*, live vaccine strain | A. Sjöstedt |
|  | LVS∆*ftrC* | | LVS with deletion of *ftrC* | This study |
|  | LVS/p*ftrC*+ | | LVS with plasmid *p6ftrC* over-expressing FtrC | This study |
|  | LVS/p6 | | LVS with plasmid pFNLTP6 | This study |
|  | LVS∆*hfq* | | LVS with deletion of *hfq* | [1] |
|  |  | |  |  |
| *E. coli* | | |  |  |
|  | DH5α | | cloning vector | Strain collection |
|  | ER2566 | |  | New England Biolabs |
|  |  | |  |  |
| **Plasmid** | | |  |  |
|  | pCR2.1-TOPO | | PCR cloning vector, Km^R^, Amp^R^ | Invitrogen |
|  | pMP812 | | *sacB* suicide vector, Km^R^ | [2] |
|  | pMP-Δ*ftrC* | | pMP812 containing ~2 kb fragment for deletion of *ftrC* | This study |
|  | pMP-Δ*FTL_1293* | | pMP812 containing ~2 kb fragment for deletion of *FTL_1293* | This study |
|  | pFNLTP6 | | *F. tularensis* plasmid, Km^R^ | [3] |
|  | p6*ftrC* | | Plasmid p6bfr with *ftrC* under control of strong Pbfr promoter | This study |
|  | p6*ftrB* | | Plasmid p6bfr with *ftrB* under control of strong Pbfr promoter | This study |
|  | pTYB21 | | Expression vector with T7 promoter and Intein Tag | New England Biolabs |
|  | pTYB21-Hfq_FTU_ | | pTYB with *F. tularensis hfq* gene , creating N-terminal Intein fusion | This study |
|  |  | |  |  |
| **Primer** | | |  |  |
|  | 3’ adapter | | P-^-^rUrUrUCGGGCCGCGGACTGTidT | [4], cDNA cloning, RACE |
|  | 5’ adapter | | GATATGCGCGAATTCCTGTAGAACGAACACTAGGGGrArArA | [4], cDNA cloning, RACE |
|  | 5’PRIMER | | GTAGAACGAACACTAGGGGAAA | [4], cDNA cloning, RACE |
|  | 3’PRIMER | | GACAGTCCGCGGCCCGAAA | [4], cDNA cloning, RACE |
|  | GSP_5’RACE | | GTCAACCCGTTATGATATATAAACC | 5’ RACE |
|  | GSP_3’RACE | | GGGGCAATCCCTTGTGGTTGCC | 3’ RACE |
|  | ftrC_DelR | | gcGCGGCCGCCTAGTATTATCAATTGGGTAAAAAGG | Deletion *ftrC* |
|  | ftrC_DelB | | GTAGGGGTTGGTTTTATGCCAACCC | Deletion *ftrC* |
|  | ftrC_DelO | | GGGTTGGCATAAAACCAACCCCTACCCATTTTTTGCATTTTTTATTCTTCTAATTCAC | Deletion *ftrC* |
|  | ftrC_DelT | | gcGTCGACCACAGGTATCTGTGGCTTTGTAGCC | Deletion *ftrC* |
|  | ftrC_DelCheck1 | | GATGATAAAAAATAATATTTAACAAATATC | Verification of *ftrC* deletion |
|  | ftrC_DelCheck2 | | GACCAATCTTTAGGTTTAAAAGCC | Verification of *ftrC* deletion |
|  | ftrC_DelCheck3 | | GAAAATTCAAATGCAAAAATTGAAAAAGC | Verification of *ftrC* deletion |
|  | ftrC_DelCheck4 | | GTTATTTAGCAAATGGCTTTCTAAATTCTCGG | Verification of *ftrC* deletion |
|  | Probe C | | GCGGTTGACCTGTGTGTCAACCCG | Northern blot probe FtrC |
|  | Probe 5S | | cggcgttttgtagtttcacttctgagttcggaatggg | Northern blot probe 5S RNA |
|  | Pbfr_F | | gcCATATGGATGGTTACTATTGCCATCATCAC | Amplification of Pbfr promoter |
|  | Pbfr_R | | gcGCGGCCGCTTAGTATCTAATTATATATCAAAATATC | Amplification of Pbfr promoter |
|  | ftrC_ovx_F | | gcGCGGCCGCTAGGGGCAATCCCTTGTGGTTGCCC | Amplification of *ftrC* |
|  | ftrC_ovx_R | | gcGCTAGCGAAGAATAAAAAATGCAAAAAATGG | Amplification of *ftrC* |
|  | Pbfr_FE | | GGTACCATATGAATTCGATGGTTACTATTGCCATCATCAC | Amplification of Pbfr promoter |
|  | Pbfr_R_ftrB | | GTATCTAATTATATATCAAAATATCTA | Amplification of Pbfr promoter |
|  | FtrB_ovx_F | | TAGATATTTTGATATATAATTAGATACATGATACTAACTTAACGTCGGTA | Amplification of *ftrB* |
|  | FtrB_ovx_R | | GGCCGCCCGGGAATTCCTCTAGAAAGTATTAGTTAATAA | Amplification of *ftrB* |
|  | hel_F | | GGGATGTCGCCTTTTGATTTTC | [5], qRT-PCR |
|  | hel_R | | CTCTTTTGTCCCTTGTGCTTGC | [5], qRT-PCR |
|  | ftrC_F | | GGGCTAACCTATGTGTTAGCCC | qRT-PCR |
|  | ftrC_R | | GCGGTTGACCTGTGTGTCAACCCG | qRT-PCR |
|  | *FTL_1293_*F | | tcaaaccaagaatacctgaatctgga | qRT-PCR |
|  | *FTL_1293_*R | | cgagctcttggttgctggcttgt | qRT-PCR |
|  | *FTL_0698_*F | | caaatcacagtcccgctgagga | qRT-PCR |
|  | *FTL_0698_*R | | gccgcttgagcaagaacaaagc | qRT-PCR |
|  | *FTL_0880_*F | | caagatggccacgcagaagc | qRT-PCR |
|  | *FTL_0880_*R | | ccaggtgttccatctggcattt | qRT-PCR |
|  | *FTL_0881_*F | | tctgatattctaaatgctgtaaaagatggaaa | qRT-PCR |
|  | *FTL_0881_*R | | aattgcccagatacttttatctctggttt | qRT-PCR |
|  | FtrB_F | | TCGGTAGTCATTCCCGCGTA | qRT-PCR |
|  | FtrB_R | | CAATCCTCGTGCTTGACACA | qRT-PCR |
|  | T7_ftrC_F | | GGGGGAATTCTAATACGACTCACTATAGGGGCAATCCCTTGTGGTTGCCC | *in vitro* transcription, FtrC |
|  | T7_ftrC_R | | AAAAAATGCAAAAAATGGTATAAAAGGGC | *in vitro* transcription, FtrC |
|  | T7_FTL_1293_F | | GGGGGAATTCTAATACGACTCACTATAGGGAACATTTATAAGTACTGTTTTGTGGTATG | *in vitro* transcription, FTL_1293 |
|  | T7_FTL_1293_R | | CTAAAATATTTTTGAATCAAATATAGCTAG | *in vitro* transcription, FTL_1293 |
|  | T7_FTL_1293short_R | | CTTATGAAACCTTGATCTCTAAGTGCTCTACG | *in vitro* transcription, FTL_1293* |
|  | Hfq-IMPACT-Up | | GCCAGAACGGAAGAGCTATGTCAAGAATATCATCTTTACAAGACCCGTTC | Amplification of *hfq* |
|  | Hfq-IMPACT-Down | | GCGAATTCGATATATTACTCGTGAATATTACCTTCATTC | Amplification of *hfq* |
|  | 1293_AF | | GGCGTCGACTCTCGATATACTCAGTGGGAC | Deletion of *FTL_1293* |
|  | 1293_AR | | ACCACAAAACAGTACTTATAAATG | Deletion of *FTL_1293* |
|  | 1293_BR | | CATTTATAAGTACTGTTTTGTGGTGCTTGTTAGAGATTTTATAATTAAC | Deletion of *FTL_1293* |
|  | 1293_BR | | GGCGGATTCGCGATAATTATTCAGAAATATAGTTCC | Deletion of *FTL_1293* |
|  | 1293_up | | GTGGGAGCTGTAATTTCACC | Verification of *FTL_1293* deletion |
|  | 1293_down | | TTTGAATTAATCCTTGGACCTG | Verification of *FTL_1293* deletion |
|  |  | |  |  |

^a^ Bases preceded by r designates a ribonucleotide whereas all other are deoxyribonucleotides. idT designates an inverted deoxythymidine. P designates a phosphorylated 5’ end.

**References**

1. Meibom KL, Forslund AL, Kuoppa K, Alkhuder K, Dubail I, et al. (2009) Hfq, a novel pleiotropic regulator of virulence-associated genes in *Francisella tularensis*. Infect Immun 77: 1866-1880.

2. LoVullo ED, Molins-Schneekloth CR, Schweizer HP, Pavelka MS, Jr. (2009) Single-copy chromosomal integration systems for *Francisella tularensis*. Microbiology 155: 1152-1163.

3. Maier TM, Havig A, Casey M, Nano FE, Frank DW, et al. (2004) Construction and characterization of a highly efficient *Francisella* shuttle plasmid. Appl Environ Microbiol 70: 7511-7519.

4. Postic G, Frapy E, Dupuis M, Dubail I, Livny J, et al. (2010) Identification of small RNAs in *Francisella tularensis*. BMC Genomics 11: 625.

5. Brotcke A, Weiss DS, Kim CC, Chain P, Malfatti S, et al. (2006) Identification of MglA-regulated genes reveals novel virulence factors in *Francisella tularensis*. Infect Immun 74: 6642-6655.
